# Change in Reciprocal Inhibition of the Forearm with Motor Imagery among Patients with Chronic Stroke

**DOI:** 10.1155/2018/3946367

**Published:** 2018-04-29

**Authors:** Michiyuki Kawakami, Kohei Okuyama, Yoko Takahashi, Miho Hiramoto, Atsuko Nishimura, Junichi Ushiba, Toshiyuki Fujiwara, Meigen Liu

**Affiliations:** ^1^Department of Rehabilitation Medicine, Keio University School of Medicine, Tokyo, Japan; ^2^Tokyo Bay Rehabilitation Hospital, Chiba, Japan; ^3^Department of Biosciences and Informatics, Faculty of Science and Technology, Keio University, Kanagawa, Japan; ^4^Keio Institute of Pure and Applied Sciences (KiPAS), Kanagawa, Japan; ^5^Department of Physical medicine and Rehabilitation, Juntendo University Graduate School of Medicine, Tokyo, Japan

## Abstract

We investigated cortically mediated changes in reciprocal inhibition (RI) following motor imagery (MI) in short- and long(er)-term periods. The goals of this study were (1) to describe RI during MI in patients with chronic stroke and (2) to examine the change in RI after MI-based brain-machine interface (BMI) training. Twenty-four chronic stroke patients participated in study 1. All patients imagined wrist extension on the affected side. RI from the extensor carpi radialis to the flexor carpi radialis (FCR) was assessed using a FCR H reflex conditioning-test paradigm. We calculated the “MI effect score on RI” (RI value during MI divided by that at rest) and compared that score according to lesion location. RI during MI showed a significant enhancement compared with RI at rest. The MI effect score on RI in the subcortical lesion group was significantly greater than that in the cortical lesion group. Eleven stroke patients participated in study 2. All patients performed BMI training for 10 days. The MI effect score on RI at a 20 ms interstimulus interval was significantly increased after BMI compared with baseline. In conclusion, mental practice with MI may induce plastic change in spinal reciprocal inhibitory circuits in patients with stroke.

## 1. Introduction

Motor imagery (MI) is the internal representation of an action without engaging in its actual physical execution. Neuroimaging findings indicated the activation of overlapping brain areas during MI and motor execution of the same task. [1–8]. Facilitation of the corticospinal descending volley during MI is nonetheless specific to the prime agonist muscles of the imagined task [9–12]. Reduced intracortical inhibition may cause corticospinal facilitation during MI [13, 14].

Mental practice (MP) has been popularized as a mental training intervention in which individuals imagine performing a given task. MP is a process in which an individual repeatedly mentally rehearses an action or a task (i.e., MI) without actually physically performing the action or task. MI has been used to specifically describe this mental task [15]. Several researchers have reported that MP combined with MI is a useful strategy for repetitive practice and skill learning, including for the paretic arm after stroke [16–20].

Despite its clinical promise, few studies have examined MP mechanisms. Some researchers reported that a rehabilitative program for the affected arm that incorporates MP appears to induce significant cortical reorganization as assessed with functional magnetic resonance imaging (MRI) [21, 22]. Few reports have described changes in spinal pathways after MP.

Reciprocal inhibition (RI) is a term that describes the inhibition of antagonist neuron pools immediately prior to or during activity within an agonist muscle [24, 25]. Voluntary muscle contraction is linked to proportional inhibition of its antagonist [25, 26]. Patients with stroke show reduced or absent RI of the forearm from the extensor carpi radialis (ECR) to the flexor carpi radialis (FCR) on the affected side [27]. In the lower limb, supraspinal input from the motor cortex plays an important role in modulating RI [28–33]. The mechanisms of supraspinal modulation are thought to involve spinal Ia inhibitory interneurons that receive descending input from the motor cortex via corticospinal pathways [34].

We hypothesized that MI of wrist extension on the affected side is a potential new strategy for modulating RI of the forearm in patients with stroke. However, to the best of our knowledge, no reports have investigated changes in RI during MI or after MI training. The goals of this study were (1) to describe RI during MI in patients with chronic stroke and (2) to examine the change in RI after MP using a brain-machine interface (BMI) system. Thus, we investigated the “mechanisms,” that is, cortically mediated changes in RI following MI in short- and long(er)-term periods.

## 2. Materials and Methods

### 2.1. Study 1: RI during MI in Stroke Patients

#### 2.1.1. Participants

The experiments were carried out with 24 stroke patients (aged 22–68 years). Criteria for inclusion in the study were (1) the time since stroke onset was longer than 150 days; (2) no cognitive deficits; (3) no pain in the paretic upper extremity; (4) passive extension range of motion greater than 0 degrees of the affected wrist and −10 degrees of metacarpophalangeal joints; and (5) no severe proprioceptive deficits in the affected upper extremity.

The mean age of the study sample was 50.1 years, and the median time since stroke onset was 1099 days (range, 259 to 4467 days). Clinical details of the participants are shown in Table 1. The purpose and procedures of the study were explained to the participants, and informed consent was obtained. The study was approved by the institutional ethics review board and registered to the UMIN Clinical Trial Registry (UMIN000001986).

#### 2.1.2. Assessment

*(1) Clinical Evaluations*. Stroke type (ischemic or hemorrhagic) and stroke location were confirmed with either MRI or computed tomography imaging.

The Stroke Impairment Assessment Set (SIAS) motor test and Fugl-Meyer Assessment (FMA) were used as measures of motor function in the affected upper extremity. The SIAS is a standardized measure of stroke impairment consisting of 22 subcategories [35, 36]. The paretic side motor functions of the upper extremity are tested with the knee-mouth test and the finger test. They are rated from 0 to 5, in which 0 indicates the most severe paralysis and 5 indicates no paresis. In addition, the score of 1 for the finger test is divided into three subscales: 1A (mass flexion), 1B (mass extension), and 1C (minimal individual movement). The FMA is a commonly used measure with excellent interrater reliability and construct validity [37–39]. The FMA consists of test A (shoulder/elbow/forearm: 36 points, A score), test B (wrist: 10 points, B score), test C (hand/finger: 14 points, C score), and test D (coordination: 6 points, D score).

The modified Ashworth scale (MAS) was used to assess spasticity in the affected upper extremity [40]. To determine sensory function, the SIAS sensory function was used [35, 36]. The paretic side position sense of the upper extremity was tested with the index finger or thumb movement. The score was graded in four grades from 0 to 3. When the patient detected no position change after the maximum possible passive motion of the index finger or thumb, a score of 0 was given. A score of 1 means that the patient could recognize movement of the digits but not the correct direction, even at maximal excursion. When the patient could correctly perceive the direction of a moderate excursion, the score was 2. A score of 3 means that the patient correctly identified the direction of a slight movement.

*(2) H Reflex and RI*. The participants were seated in a comfortable chair with their affected arms supported and relaxed on the armrests in pronation. The angle of their elbows was kept at 70–90 degrees. Percutaneous electrical pulses of 1 ms duration at a frequency of 0.3 Hz were delivered through surface electrodes. H reflexes were recorded from the FCR muscle in the paretic arm of patients with stroke following submaximal electrical stimulation of the median nerve at the antecubital fossa. The reflex responses were measured as the peak-to-peak amplitude of the H reflex recorded with a bipolar disc electrode placed over the FCR muscle [23].

RI was assessed using an FCR H reflex conditioning-test paradigm [23]. Ten conditioned and 10 test H reflexes were averaged at each time point. The test FCR H reflex amplitude was maintained at 15–20% of the maximal M wave amplitude for each block trial. Conditioning stimulation to the radial nerve was delivered at the spiral groove. Stimulus intensity of the conditioning stimulation was 1.0 motor threshold, which was defined as a 100 *μ*V response of the ECR muscle. The conditioning test interstimulus interval (ISI) was set at two intervals of 0 and 20 ms based on previous reports [41–46]. The first phase, that is, ISI of 0 ms, is related to the Ia disynaptic pathway [23]. The second inhibitory phase, ISI of 20 ms, is thought to represent presynaptic inhibition [47]. The size of the conditioned H reflex was expressed as a percentage of the size of the unconditioned H reflex at each interval (e.g., RI 0 ms = conditioned H reflex amplitude of the ISI at 0 ms/test H reflex amplitude × 100).

In addition, participants were asked to imagine wrist extensions of their paretic wrist during assessment of RI as mentioned above. When the participant imagined wrist extensions of their paretic wrist, we checked the electromyographic activity of the ECR muscle. Thus, we assessed two patterns of RI. One was RI at rest, and the other was RI during MI. We calculated the “MI effect score on RI,” which was the value of RI during MI divided by the value of RI at rest expressed as a percentage. That is, if MI led to a strong RI, the MI effect score was smaller and less than 100%.

#### 2.1.3. Statistical Analyses

Comparison between the conditioned H reflex amplitude and test H reflex amplitude at each ISI was performed using the paired *t*-test. We compared RI at rest and during MI for each ISI group (ISI 0 ms, 20 ms) with the Wilcoxon signed-rank test and set the significance level at less than 0.05. Effect sizes were calculated using Cohen's *d* statistics, and the magnitude of the group difference was defined as small if *d* = 0.2, medium if *d* = 0.5, or large if *d* = 0.8, considering the clinical significance of the variables.

Patients were divided into two additional groups (cortical lesion group, subcortical lesion group) according to the stroke location. We compared the RI at rest and during MI for the two groups according to the stroke location using Welch's *t*-test and set the significance level at 0.05.

### 2.2. Study 2: The Change in RI during MI after MI Training Using the BMI in Stroke Patients with Severe Hemiparesis

#### 2.2.1. Participants

Participants were recruited from an outpatient rehabilitation clinic of a university hospital. Patients were included in the study if they met the following criteria: (i) first unilateral subcortical stroke not involving the sensorimotor cortex as confirmed by brain MRI or computed tomography; (ii) time since stroke onset of more than 180 days; (iii) ability to raise the paretic hand to the height of the nipple; (iv) inability to extend the paretic fingers; (v) no motor improvement during the 30 days prior to starting the intervention as confirmed by both the patients and their physicians; (vi) ability to walk independently in their daily lives; (vii) no severe cognitive deficits as determined by a Mini Mental State Examination score > 25; (viii) no severe pain in the paretic upper extremity; (ix) no pacemaker or other implanted stimulator; and (x) no history of seizures within the past 2 years and no use of anticonvulsants 1 month before the intervention.

From January 2013 to March 2014, 11 patients were enrolled in the study. The study purpose and procedures were explained to the participants, and written informed consent was obtained from each.

The mean age of the study sample was 50.6 years (SD 10.9), and the median time since stroke onset was 30.5 months (range, 9 to 180 months). Clinical details of the participants are shown in Table 2.

This study was approved by the institutional ethics review board. This study was registered as a clinical trial with the University Hospital Medical Information Network in Japan (UMIN Critical Trial Registry UMIN000008468).

#### 2.2.2. Intervention

*(1) Electroencephalographic Recording*. The participants wore a headset with two brush-type electrodes [48]. Electroencephalography was recorded with Ag-AgCl electrodes (1 cm in diameter), at C3 and the left ear in patients with right hemiparesis and at C4 and the right ear in patients with left hemiparesis, according to the international 10–20 system. An additional electrode was placed at a position 2.5 cm anterior to C3 or C4. A ground electrode was placed on the forehead, and the reference electrode was placed on either A1 or A2 (ipsilateral to the affected hemisphere). The experimenter monitored the electroencephalographic waveform on the computer at all times during BMI training.

*(2) Event-Related Desynchronization (ERD) Quantification*. As a feature to enhance the excitability of the ipsilesional sensorimotor cortex, ERD, which is a diminution of the alpha band (8–13 Hz) of the mu rhythm amplitude, was calculated as follows. ERD was used as a trigger signal for the feedback system in BMI training. ERD was expressed as the percentage of the power decrease related to the 1 s reference interval before the direction of intention. ERD at a certain frequency was calculated for each time and frequency according to the following equation:

(1) $$\begin{array}{c} ERD\;\left(f,t\right)=\left\{\frac{\left(R\left(f\right)-A\left(f,t\right)\right)}{R\left(f\right)}\right\}\times 100\left(\%\right), \end{array}$$

where *A*(*f*, *t*) is the power spectrum density of electroencephalography at a certain frequency band *f* (Hz) and time *t* (s) since the imagery task was started, and *R*(*f*) is the power spectrum at the same frequency *f* (Hz) of the baseline period.

*(3) BMI Training*. MI-based BMI training was performed for approximately 45 min a day, 5 times a week, for a total of 10 days. All participants underwent 40 min of standard occupational therapy per day, which consisted of gentle stretching exercises, active muscle reeducation exercises, and introduction to bimanual activities in their daily lives.

Because the details of the training protocol are explained elsewhere [48], a brief overview is described here. The participants were seated in a comfortable chair with their arms supported and relaxed on the armrest in pronation. The motor-driven orthosis was attached to the paretic hand to achieve finger extension movement at the metacarpophalangeal and proximal interphalangeal joints.

Participants faced a 15.4-inch computer monitor placed approximately 60 cm in front of their eyes, and pegs were set on the desk peg board next to the computer. Participants were asked to pick up a peg with the paretic hand with the orthosis.

A star-shaped cursor began to move at a fixed rate from left to right across the computer monitor over an 8 s period. Participants were instructed to rest for 5 s and then to imagine extending their paretic fingers for the next 3 s, depending on the task cue from the monitor. If the mu ERD was detected after the cue instruction of MI, the star-shaped cursor moved down on the screen as visual feedback, and then the motor-driven hand orthosis moved as the orthosis extended the paretic fingers. If the mu ERD was not detected after the cue, which meant that MI was not successfully performed, the orthosis did not move.

#### 2.2.3. Assessment

We assessed the following items before and after BMI training: RI at rest, RI during MI, MI effect score on RI, and FMA.

#### 2.2.4. Statistical Analyses

The Wilcoxon signed-rank test was used to compare the FMA score, RI at rest, and MI effect score on RI with a between-subject factor of time (pre- and post-BMI training). The significance level was set at 0.05.

## 3. Results

### 3.1. Study 1: RI during MI in Stroke Patients

The mean conditioned H reflex amplitude at the ISI of 0 ms was 1.06 ± 0.69 mV, which was significantly smaller than the test H reflex amplitude that was 1.49 ± 0.79 mV (*p* < 0.001). Similarly, the conditioned H reflex amplitude at the ISI of 20 ms was 1.33 ± 0.64 mV, which was significantly smaller than the test H reflex amplitude (1.43 ± 0.70 mV) (*p* < 0.001).

**Figure 1:**
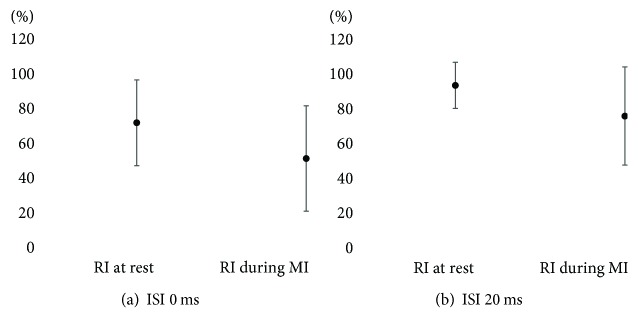
Comparison between reciprocal inhibition at rest and reciprocal inhibition during motor imagery. Significant changes were found in reciprocal inhibition (RI) during motor imagery (MI) at both an interstimulus interval (ISI) of 0 ms and 20 ms compared with RI at rest. Data are the mean ± standard deviation.

**Figure 2:**
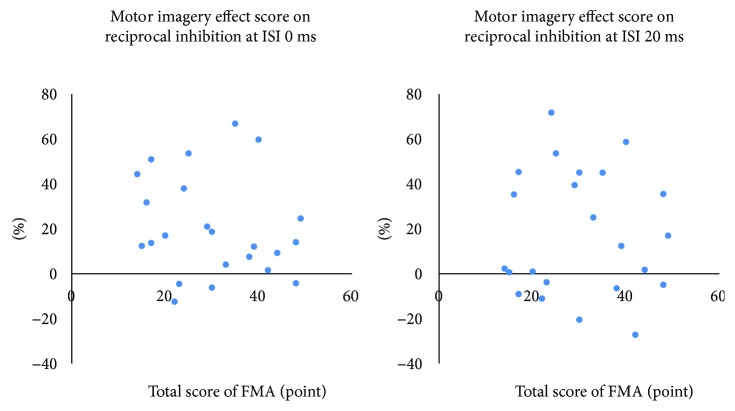
Correlation between the motor imagery effect score on reciprocal inhibition and motor function in the affected upper extremity. With a conditioning test interstimulus interval (ISI) of both 0 and 20 ms, no significant correlation was found between the motor imagery (MI) effect score on reciprocal inhibition (RI) or the motor function in the affected upper extremity as assessed with the Fugl-Meyer Assessment (FMA).

The Wilcoxon signed-rank test showed significant enhancement in RI during MI both at an ISI of 0 ms and 20 ms compared with RI at rest (71.20 ± 24.68 to 51.13 ± 30.36 and 93.44 ± 13.28 to 75.79 ± 28.21, resp.) (*p* < 0.01) (Figure 1). Cohen's *d* statistics for the RI at an ISI of 0 ms and 20 ms were 0.74 and 0.80, respectively. No relationship was observed between the MI effect score on RI and FMA (Figure 2).

Regarding the stroke lesion, we observed no significant differences between the RI at rest and during MI for either ISI in the cortical lesion group. On the other hand, significant differences were observed between the RI at rest and during MI in the subcortical lesion group. In the subcortical lesion group, MI enhanced the RI for both ISIs (Table 3).

### 3.2. Study 2: The Change in RI during MI after MI Training Using BMI in Stroke Patients with Severe Hemiparesis

The Wilcoxon signed-rank test showed significant improvement in FMA after BMI training (21.4 ± 5.5 before versus 26.3 ± 4.9 after training, *p* < 0.001).

Although we found no differences in RI at rest between before and after BMI, the MI effect score on RI at an ISI of 20 ms was significantly increased after BMI (Table 4). MP using BMI was thought to enhance the modifying effect of MI on RI.

## 4. Discussion

This is the first study to show that RI of the antagonist muscle was increased during MI in patients with stroke. Moreover, this change was not found in patients whose lesion was in the cerebral cortex. In addition, we demonstrated that RI was reinforced by MI training using BMI technology. These results are helpful for understanding the effect of MI on spinal neural circuits.

A lot of electrophysiological research investigating the excitability of cortical and spinal pathways during MI has been performed. In previous research using transcranial magnetic stimulation, many researchers showed that the excitability of the corticospinal tract is increased during MI [9–12]. Therefore, MI-induced modulation is considered to occur at cortical levels. However, little is known about the effect of MI on spinal neural circuits, which has been measured with several methods (i.e., H reflex, F wave, cervicomedullary stimulation, and motor evoked potential). Cervicomedullary stimulation-evoked potentials, which provide a direct measurement of motoneuron excitability by eliciting a single volley in descending axons at the pyramidal decussation, are increased during MI [49]. Moreover, the frequency of F wave occurrence, in which F waves are produced by backfiring of alpha motor neurons, is also increased during MI [50, 51]. Thus, MI may generate a subliminal impulse that does not induce a discharge of alpha motor neurons. The result in this study indicated that not only alpha motor neurons but also interneurons at the spinal level were modulated during MI, because we observed an increase in disynaptic and presynaptic inhibition of agonist muscles during MI in patients with stroke. These modulations are similar to those seen in motor execution [52]. In previous studies, the authors thought that RI of the antagonist muscles may occur at the cortical and spinal levels when measurements were performed using indirect methods such as transcranial magnetic stimulation and H reflex during MI [53, 54]. In our study, we directly showed that RI of the antagonist muscle was increased at the spinal level using the FCR H reflex conditioning-test paradigm during MI in patients with stroke.

The amount of RI, especially presynaptic inhibition, was different depending on whether the lesion included the cerebral cortex. These results implied that patients who had brain lesions that included the cerebral cortex could not sufficiently modulate their spinal neural circuits during MI. Several hypotheses may explain this observation. The first hypothesis is that the exercise image is not performed well by patients with cortical lesions. The mechanisms of impairments in MI performance have not been clarified. MI is the internal representation of an action without any overt motor output. Therefore, its origin is an internal process at the level of the cortex. Indeed, parietal lobe damage impairs MI performance [55, 56]. In this study, most patients in the cortical lesion group had substantial damage to the parietal lobe because of cerebral infarction at the middle cerebral artery. Although we did not precisely investigate the damaged area, we speculate that patients in the cortical lesion group could not perform kinesthetic MI correctly. Therefore, the difference in the MI effect score on RI between the cortical lesion group and the subcortical lesion group may reflect the vividness of MI. This finding should be verified in a larger within-subgroup sample in a future study.

A second hypothesis is also possible. Previous studies have shown that presynaptic inhibition at the spinal level is cortically mediated [57–60]. Thus, lesions in cerebral sites may prevent cortically mediated changes in the inhibitory mechanisms that take place at the spinal level. This hypothesis is consistent with the results of our study 2. Presynaptic inhibition during MI was reinforced by MI training using BMI technology in patients with subcortical stroke in which the sensorimotor cortex was spared. Because presynaptic inhibition during the rest condition was not changed, we consider that plastic changes did not occur at lower nervous system levels that are intrinsically involved with RI. The MI-induced descending volley to the interneurons involved with presynaptic inhibition may have increased after BMI rehabilitation. In the BMI training, mu ERD was used as a biomarker of motor intention. In a previous study, Takemi et al. reported that the amount of ERD during MI is associated with corticospinal excitability and the potentiation of spinal motoneurons [14, 50]. ERD during MI is gradually increased in consecutive BMI rehabilitation sessions [61]. Thus, the MI-induced descending volley that is enhanced by the effect of BMI rehabilitation may increase presynaptic inhibition during MI. This result supports the hypothesis that presynaptic inhibition at the spinal level is cortically mediated.

Our study has some limitations. First, the small sample size is a limitation, and some variables may have shown no significant differences between groups because of the small sample size. The unbalanced sample with regard to stroke severity is also a limitation. This study sample did not include patients who have fairly mild paresis. Third, the ability to perform MI and the quality of MI were not evaluated. In future studies, the ability to perform MI should be assessed with a questionnaire (e.g., the Kinesthetic and Visual Imagery Questionnaire). Despite these limitations, we believe that the present findings are helpful for understanding the effect of MI on spinal neural circuits and are also useful as supplementary evidence about the effectiveness of MI training in patients with stoke.

## 5. Conclusion

Our findings indicate that RI of the antagonist muscle was increased while imagining a contraction of the agonist muscle in patients with stroke and that RI was reinforced by MI training using BMI training.

## Figures and Tables

**Table 1:** Clinical details of participants in study 1.

Age (years)	Diagnosis	Stroke location	Paretic side	TFO (days)	SIAS		MAS		
					Finger	Knee-mouth	Finger	Wrist	Elbow
65	CI	Medulla	Lt	516	1B	3	2	1+	1+
50	CI	Thalamus	Rt	584	1B	3	2	1+	1+
50	CI	MCA	Rt	4467	1C	3	1	1+	1+
43	CH	Putamen	Rt	829	1A	4	1	1	1+
39	CH	Putamen	Rt	358	1B	4	1	1	1+
63	CI	Insular cortex	Lt	863	1A	2	1+	2	1+
42	CH	Putamen	Rt	849	1A	4	2	1+	1+
49	CH	Putamen	Rt	343	1C	3	1	0	1
46	CH	Putamen	Lt	2109	1A	3	1	1	1
39	CI	MCA	Lt	687	1B	4	1	1	1
43	CI	Pons	Lt	668	1C	3	1+	2	1+
61	CI	Internal capsule	Rt	259	1B	3	1+	1	1+
33	CI	MCA	Rt	649	1A	3	1+	1+	1+
77	CH	Putamen	Rt	535	1C	3	1	0	1
46	CH	Putamen	Lt	1958	1A	3	1	1	1+
42	CH	Putamen	Lt	499	1A	3	2	2	1+
50	CI	MCA	Rt	1525	1A	3	1+	1+	1
55	CH	Thalamus	Lt	1922	1A	2	1+	0	0
37	CH	Putamen	Lt	1101	1A	2	2	1	1+
60	CH	Putamen	Rt	1146	1A	3	1	0	1
68	CI	Corona radiata	Lt	1386	1C	3	1	1+	1+
50	CH	Putamen	Rt	313	1A	2	1+	1	1+
51	CI	MCA	Lt	2160	1C	3	2	1+	2
43	CI	MCA	Lt	621	1A	2	1	1+	1

TFO: time from onset; SIAS: Stroke Impairment Assessment Set; MAS: modified Ashworth scale; CI: cervical infarction; CH: cervical hemorrhage; MCA: middle cerebral artery.

**Table 2:** Clinical details of participants in study 2.

Age (years)	Diagnosis	Stroke location	Paretic side	TFO (days)	SIAS		MAS		
					Finger	Knee-Mouth	Finger	Wrist	Elbow
46	CH	Putamen	Rt	1958	1A	3	1	1	1+
42	CH	Putamen	Rt	499	1A	3	2	2	1+
53	CH	Putamen	Rt	385	1A	3	2	1+	1+
50	CI	MCA	Lt	1525	1A	3	1+	1+	1
55	CH	Thalamus	Rt	1922	1A	2	1+	0	0
37	CH	Putamen	Rt	1101	1A	2	2	1	1+
47	CI	Putamen	Lt	410	1A	2	1	1+	1
60	CH	Putamen	Lt	1146	1A	3	1	0	1
65	CI	Corona radiata	Lt	695	1A	3	1	0	0
51	CH	Putamen	Lt	1522	1A	3	2	3	2
53	CI	MCA	Lt	983	1A	2	1	2	0

TFO: time from onset; SIAS: Stroke Impairment Assessment Set; MAS: modified Ashworth scale; MCA: middle cerebral artery.

**Table 3:** Reciprocal inhibition at rest and during motor imagery in the two groups according to stroke location.

	RI at rest	RI during MI	
Cortical lesion (*N* = 7)			
ISI 0 ms	70.03 ± 31.07	62.71 ± 31.08	*p* = 0.12
ISI 20 ms	96.27 ± 8.58	95.90 ± 17.40	*p* = 0.95
Subcortical lesion (*N* = 16)			
ISI 0 ms	72.42 ± 21.24	46.07 ± 28.61	*p* < 0.01
ISI 20 ms	92.20 ± 14.70	66.99 ± 27.52	*p* < 0.01

RI: reciprocal inhibition; MI: motor imagery; ISI: interstimulus interval.

**Table 4:** Motor imagery effect on reciprocal inhibition after brain-machine interface training.

	Pre-BMI	After BMI	
RI at rest			
ISI 0 ms	70.06 ± 24.46	74.71 ± 31.65	*p* = 0.47
ISI 20 ms	84.64 ± 11.47	86.53 ± 16.90	*p* = 0.73
Motor imagery effect score on RI			
ISI 0 ms	92.83 ± 58.40	47.09 ± 22.16	*p* = 0.08
ISI 20 ms	83.69 ± 24.43	66.43 ± 19.65	*p* = 0.04

BMI: brain-machine interface training; RI: reciprocal inhibition; ISI: interstimulus interval.

## Supplementary Materials

Supplementary Materials Supplementary Table 1: the result of MI effect on RI in the two groups divided by the severity of position sense, spasticity, and stroke location.
