# Supplementary material for: Change in Reciprocal Inhibition of the Forearm with Motor Imagery among Patients with Chronic Stroke
Source: Neural Plast. 2018 Apr 29;2018:3946367. doi: 10.1155/2018/3946367 (PMC5949151; doi:10.1155/2018/3946367)
Supplement: Supplementary Materials — Supplementary Table 1: the result of MI effect on RI in the two groups divided by the severity of position sense, spasticity, and stroke location. [file 3946367.f1.doc]

Supplementary Table 1. Motor imagery effect on reciprocal inhibition in the two groups divided by the severity of position sense, spasticity, and stroke location.

|  | Motor image effect score on RI ISI 0ms | Motor image effect score on RI ISI 20ms |
| --- | --- | --- |
| Sensory disturbance group (N=11) | 63.00±25.59 | 79.00±27.13 |
| Normal sensory group(N=12) | 75.96±29.25 | 84.21±31.47 |
|  | p=0.29 | p=0.69 |
|  |  |  |
| Severe Spasticity (N=12) | 82.01±25.54 | 89.92±25.31 |
| Mild Spasticity (N=11) | 56.41±24.94 | 72.77±31.27 |
|  | p=0.030 | p=0.18 |
|  |  |  |
| Cortical lesion (N=7) | 86.35±15.68 | 99.56±15.27 |
| Subcortical lesion (N=16) | 62.51±29.52 | 73.91±30.92 |
|  | P=0.027 | P=0.020 |

RI; Reciprocal inhibition.

The patients were divided into two groups (sensory disturbance group, normal sensory group) according to the severity of paretic side position sense. Patients with 3 points on the SIAS sensory function score were classified as the “normal sensory group,” and the other patients were classified as the “sensory disturbance group”. Patients were also divided into two other groups (severe spasticity group, mild spasticity group) according to the severity of wrist flexor spasticity on the paretic side. Patients who scored 1+, 2, 3, or 4 by the MAS were classified as the “severe spasticity group,” and patients who scored 0 or 1 were classified as the “mild spasticity group”. Finally, patients were also divided into two additional groups (cortical lesion group, subcortical lesion group) according to the stroke location.

We compared the MI effect score on RI for the two groups divided by the severity of position sense, spasticity, and stroke location using Welch's t test and set the significance level at less than 0.017 (0.05/3).
